# Cocoa Bioactive Compounds: Significance and Potential for the Maintenance of Skin Health

**DOI:** 10.3390/nu6083202

**Published:** 2014-08-11

**Authors:** Giovanni Scapagnini, Sergio Davinelli, Laura Di Renzo, Antonino De Lorenzo, Hector Hugo Olarte, Giuseppe Micali, Arrigo F. Cicero, Salvador Gonzalez

**Affiliations:** 1 Department of Medicine and Health Sciences, University of Molise, Campobasso 86100, Italy; E-Mail: s.davinelli@gmail.com; 2 Inter-University Consortium “SannioTech”, Benevento 82030, Italy; 3 Division of Human Nutrition, Department of Neuroscience, University of Rome Tor Vergata, Rome 00173, Italy; E-Mails: Laura.di.renzo@uniroma2.it (L.R.); delorenzo@uniroma2.it (A.L.); 4 Casa Luker S.A., Bogotà DC, Colombia; E-Mail: holarte@casaluker.com.co; 5 Dermatology Clinic, University of Catania, Catania 95123, Italy; E-Mail: cldermct@nti.it; 6 Department Medical and Surgical Sciences, University of Bologna, Bologna 40138, Italy; E-Mail: arrigo.cicero@unibo.it; 7 Dermatology Service, Memorial Sloan-Kettering Cancer Center, New York, NY 10017, USA; E-Mail: gonzals6@mskcc.org; 8 Dermatology Service, Ramon y Cajal Hospital, Madrid 28034, Spain

**Keywords:** cocoa, skin, health, phytochemicals, antioxidant, anti-inflammatory

## Abstract

Cocoa has a rich history in human use. Skin is prone to the development of several diseases, and the mechanisms in the pathogenesis of aged skin are still poorly understood. However, a growing body of evidence from clinical and bench research has begun to provide scientific validation for the use of cocoa-derived phytochemicals as an effective approach for skin protection. Although the specific molecular and cellular mechanisms of the beneficial actions of cocoa phytochemicals remain to be elucidated, this review will provide an overview of the current literature emphasizing potential cytoprotective pathways modulated by cocoa and its polyphenolic components. Moreover, we will summarize *in vivo* studies showing that bioactive compounds of cocoa may have a positive impact on skin health.

## 1. Introduction

**Figure 1:**
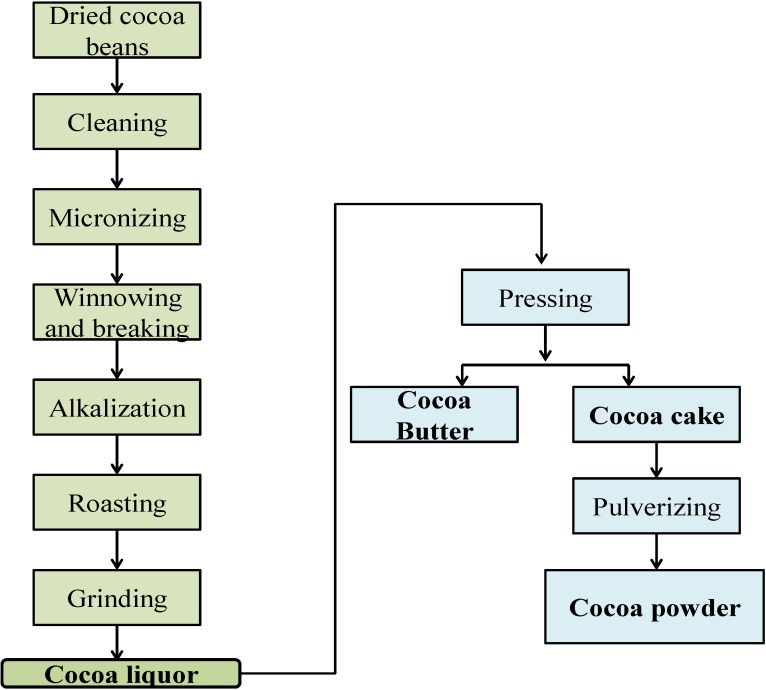
Manufacturing processes of cocoa beans.

Seeds from *Theobroma cacao* L. (Sterculiaceae) are the base for the production of the most important and widespread functional food in human history. The origin of cocoa dates back to more than 3000 years ago, and it was used for nutritional and medicinal purposes by the Mayan and Aztec civilizations [1]. Numerous reports have focused on various health-beneficial effects associated with the consumption of cocoa products [2]. Although the cocoa plant contains an enormous range of beneficial components, processing and manufacturing alters its content and bioactive moieties, particularly during roasting, fermentation and drying, as well as with the use of analytical methods for isolation, characterization and quantification of its bioactive compounds. For instance, the production of cocoa liquor (a dark brown fluid obtained by grinding cocoa nibs), which is a combination of cocoa butter and cocoa powder, involves the cleaning of the seeds followed by fermentation, drying and roasting steps. The time and temperature conditions during the roasting process affect the flavor characteristics and nutrient profile of the final product [3]. The liquor may then be processed with alkali, also known as Dutch processing or Dutching, to increase the pH and improve palatability. Furthermore, the alkalizing stage affects the chemical composition of the cocoa liquor [4]. The chemical profile of roasted cocoa beans is complex, and the primary compounds that induce its multiple beneficial functions are naturally occurring or process-derived flavonoids, theobromine and magnesium. A summary of the manufacturing steps is shown in Figure 1. The term “cocoa component” is intended to refer to a fraction derived from shell-free cocoa nib and includes chocolate liquor, partially- or fully-defatted cocoa solids, cocoa extracts, cocoa butter and cocoa nib. The potential health implications of biologically active substances present in cocoa components are well documented. Many epidemiological studies associate cocoa and chocolate consumption to a reduced risk of chronic diseases, and various health benefits of the cocoa compounds have been attributed to its antioxidant and anti-inflammatory potency [5]. In particular, the bioactive constituents of cocoa components exhibit pharmacologic effects in reducing inflammatory processes [6]. This is based on their ability to downregulate pro-inflammatory cytokines and their downstream biochemical pathways [7]. In addition, according to Lee *et al*. [8] phenolic and flavonoid contents and total antioxidant capacities of cocoa are higher than that of other phytochemical-rich foods. The antioxidant effects of the cocoa components may influence insulin resistance, reduce the risk for diabetes or stimulate redox-sensitive signaling pathways involved in the gene expression of endogenous antioxidant defenses [9]. Diet has been recognized as an important modifier of health status, and dermal health can be greatly affected by food composition. Although this is an emerging area of research and more robust findings are required, it was reported that the consumption of cocoa rich in phytochemicals may help to maintain skin health and confer photoprotection [10]. After a brief overview of typical phytochemicals present in cocoa, we will review the beneficial impact of cocoa and chocolate consumption on human health with special emphasis on skin physiology.

## 2. Cocoa Bioactive Compounds and Minerals

### 2.1. Polyphenols

Plants provide abundant minerals and phytochemicals when presented as food. Cocoa is a widely consumed food ingredient. Phytochemicals’ profile in cocoa beans varies for different cultivars and among cocoa beans and cocoa-containing foods. However, cocoa is a rich source of polyphenolic compounds with a high amount of flavonoids, specifically flavanols, also known as flavan-3-ols. Polyphenols are secondary plant metabolites that are involved in plant defense against herbivores, pathogens and ultraviolet (UV) damage [11]. More than 8000 phenolic structures are currently known, and among them, over 4000 flavonoids have been identified [12,13,14]. The basic flavonoid skeleton consists of 15 carbon atoms with two aromatic rings (Ring A and Ring B) connected by a three-carbon bridge (Ring C). Flavonoids can be divided into different sub-groups, such as anthocyanins, flavan-3-ols, flavones, flavanones and flavonols, according to hydroxylation pattern and variations in the chromane ring (Ring C) [15]. Moreover, cocoa components are particularly rich in catechins, and based on their structure, catechins are classified as flavan-3-ols. They mainly include monomeric (−) epicatechin and (+) catechin, as well as oligomeric and polymeric proanthocyanidin flavanols [5]. However, in smaller amounts, gallocatechin and epigallocatechin, have also been quantified [16]. The procyanidins represent a dominant class of proanthocyanidins, and it was found that these naturally occurring compounds are the main group responsible for the antioxidant activity of the cocoa components [17]. The bitterness is primarily due to the high levels of flavanols, and they represent a fundamental aspect of the organoleptic and palatability characteristics of chocolate [18]. More importantly, flavanols may influence healing properties and offer a wide range of health benefits. For instance, flavanol-rich cocoa consumption improves dermal blood flow, increases photoprotection and contributes to the maintenance of skin health [19,20].

### 2.2. Theobromine

In addition to polyphenols, cocoa contains methylxanthine compounds, predominantly theobromine and caffeine, but in lower amounts than those of theobromine [9]. The average contents of the individual methylxanthines is dependent on the genotype of the cacao tree. Theobromine belongs to the group of purine alkaloids, which can elicit various physiological effects, and they are synthesized in a limited number of plant species, including tea, coffee and cacao [21]. The pharmacology and toxicology of theobromine and other methylxanthines have been reviewed by Smit *et al*. [22,23]. Since theobromine has been shown to possess high bioavailability and multiple biological activities, recent cocoa intervention studies have been conducted on the ability of theobromine to increase serum HDL cholesterol [24]. Furthermore, theobromine stimulates heart muscle, relaxes bronchial smooth muscles in the lungs and plays an important role in the transmission of intracellular signals [25,26,27]. It is noteworthy that theobromine has antioxidant activity, and several antioxidant compounds may be effective treatments for depressive disorders [28]. Although new findings indicate that theobromine does not influence mood, there is a growing body of literature indicating that theobromine and cocoa flavanols, in isolation and in combination, may have measurable neurocognitive effects [29,30]. The value of antioxidants in skin health is a controversial area, but several botanical agents, including theobromine, can scavenge reactive oxygen species (ROS) generated in the skin as a consequence of UV exposure, and they can interfere with signaling pathways altered in the skin as a consequence of these ROS [31].

### 2.3. Minerals

The cocoa bean is an extremely rich source of many essential minerals, including magnesium, copper, potassium and iron. As reviewed by Steinberg *et al*. [32], most of these minerals may affect vascular health and function, improving cocoa’s nutritional effects. The predominant mineral found in cocoa is magnesium, which catalyzes a multitude of biologic reactions, including protein synthesis and energy production [32]. In addition, magnesium is an antiarrhythmic and hypotensive agent, and its deficiency has been linked to the metabolic syndrome, insulin resistance and diabetes [33,34]. Dark chocolate is also an important source of copper, and this mineral is required for processes, such as iron transport, glucose metabolism, infant growth and brain development [18,35]. Moreover, cocoa and cocoa products are rich in iron, but they are relatively low in potassium [9].

## 3. Molecular Mechanisms Relevant to Skin Health

**Figure 2:**
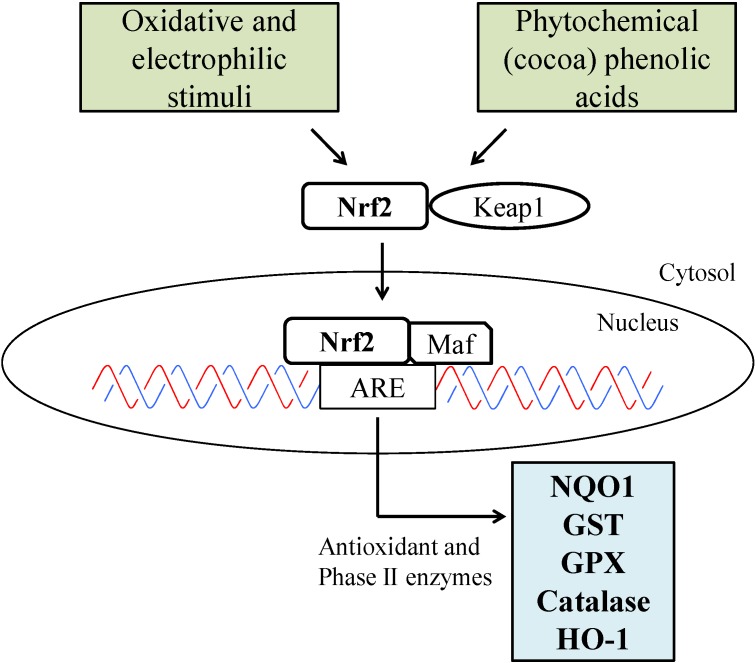
Schematic presentation of the Nrf2 pathway. When oxidants, such as ROS, RNS and dietary phytochemical compounds react with redox reactive cysteines in Keap1, Nrf2 will be released from Keap1, allowing Nrf2 to be translocated into the nucleus. In the nucleus, Nrf2 dimerizes with Maf protein and bind to ARE, which is located in the promoter of the phase II and antioxidative genes, triggering the transcription of ARE-regulated genes. Nuclear factor erythroid 2-related factor 2 (Nrf2); Kelch-like ECH-associated protein 1 (Keap1); musculoaponeurotic fibrosarcoma (Maf); antioxidant response element (ARE); NAD(P)H:quinone oxidoreductase 1 (NQO1); glutathione S-transferase (GST); glutathione peroxidase (GPX); heme oxygenase-1 (HO-1).

In recent years, there has been a growing interest, supported by a large number of experimental and epidemiological studies, for the beneficial effects of some phenolic substances, contained in commonly used spices and herbs, in preventing various age-related pathologic conditions, ranging from cancer to neurodegenerative diseases. Although the exact mechanisms by which polyphenols promote these effects remain to be elucidated, several reports have shown their ability to stimulate a general xenobiotic response in the target cells, activating multiple defense genes, activating a number of different molecular targets, impinging on several signaling pathways and showing pleiotropic activity on cells and tissues. Cocoa polyphenols, mainly flavanols in both monomeric and oligomeric form, have been shown to act as a strong antioxidants, having the potential to inhibit lipid peroxidation and to effectively intercept and neutralize ROS. In this regard, cocoa flavanols have been demonstrated to be more potent than other food polyphenols. The tricyclic structure of the flavonoids determines their antioxidant effects; phenolic quinoid tautomerism and the delocalization of electrons over the aromatic system scavenge ROS. These aromatic rings directly neutralize free radicals and chelate metals (Fe^2+^ and Cu^+^) that enhance ROS. Due to the good bioavailability, cocoa intake increases serum antioxidant capacity, protecting the endothelium from oxidative stress and endogenous ROS [36], although contemporary milk assumption decreases this ability [37]. Beyond their ROS quencher activity, cocoa polyphenols effects have been mostly associated with cocoa’s ability to interfere at a molecular level with numerous cellular antioxidant pathways. They strongly inhibit enzymes involved in ROS production. Enzymes inhibited by cocoa flavonoids include xanthene oxidase, NADPH-oxidase, tyrosine kinases and protein kinases [38]. Furthermore, cocoa flavanols have been shown to upregulate antioxidant defenses by overexpressing highly protective inducible genes involved in the cellular stress response, such as the activation of the nuclear factor erythroid 2-related factor 2 (Nrf2) signaling pathway (Figure 2). Nrf2 is a conserved master regulator of cellular antioxidant responses. Nrf2 belongs to the Cap’n’Collar family leucine zipper transcription factors and regulates the expression of genes encoding anti-oxidant and detoxifying proteins, such as glutathione S-transferase (GST), glutathione synthetase (GSS), heme oxygenase-1 (HO-1) and NAD(P)H:quinone oxidoreductase. Among the genes activated by Nrf2, HO-1 has been the object of intensive studies for its potential role in protecting several tissues against cell death [39]. Cocoa extract has been recently shown to efficiently induce HO-1 expression through the activation of Nrf2 in mice and, by this, to protect neurons against different challenges [40]. Flavonoids have a number of other properties that may contribute to their protective and healing effects, including anti-inflammatory and antiplatelet activity, immunoregulatory properties and beneficial effects on vascular endothelium. The epicatechin content of cocoa is primarily responsible for its favorable impact on vascular endothelium, which is the result of both acute and chronic upregulation of nitric oxide (NO) production [41]. NO synthesis is the most investigated endothelial function in relation to cocoa over the past 10 years, and many authors have reported that cocoa polyphenols significantly increase plasma concentrations of NO [42]. The predominant mechanistic hypothesis is that cocoa components, in particular epicatechin, stimulate endothelial nitric oxide synthases (eNOS) activity, inhibit arginase and NADPH oxidase, leading to lower levels of superoxide and, hence, higher levels of NO. Although this is not the only mechanism involved, a substantial increase in NO synthesis may account for flow-mediated dilation and lower blood pressure following intervention treatments. Cocoa procyanidins are also potent inhibitors of mitogen-activated protein kinase kinase (MEK) and membrane type-1 (MT1)-matrix metalloproteinase (MMP). They subsequently inhibit the expression and activation of pro-MMP-2, as well as the invasion and migration of human vascular smooth muscle cells (VSMCs) [43]. Both of these mechanisms have a critical relevance, not only in flavanols cardioprotective effects, but also for their potential use in cancer prevention and photoprotection. Ramiro *et al*. [44] studied the effects of a cocoa extract on the secretion and RNA expression of various proinflammatory mediators by macrophages. Of these, monocyte chemoattractant protein (MCP)-1 and tumor necrosis factor (TNF)-α were significantly and dose-dependently diminished by the extract. All cocoa flavonoids tested were capable of reducing MCP-1 secretion after 6 h of LPS activation. The regulatory effects of cocoa flavanols on nuclear factor-κB (NF-κB) activation have also been studied. (−)-Epicatechin, (+)-catechin and their dimeric forms were found to inhibit the NF-κB activation induced by phorbol esters in T-cells, with a clear reduction of NF-κB-DNA binding activity that leads to a reduction in interleukin 2 (IL-2) production. These effects were related to the direct interaction with the inhibitor of κB (IκB) to prevent its phosphorylation, thereby preventing NF-κB activation [45].

## 4. Role of Cocoa Polyphenols in the Skin: Anti-Aging Effects

Skin aging is a complex process that involves intrinsic and exogenous causes. Intrinsic skin aging is inevitable, but exogenous aging is caused by exogenous harmful environments and can be, at least partially, prevented. Photo-oxidative damage caused by solar UV light is the main cause of extrinsic aging of the skin, a phenomenon known as photoaging. UV damage can be linked mostly to the photochemical overproduction of ROS, which induces a complex molecular cascade able to activate inflammation, to accelerate physiological aging and determining a typical dermal/epidermal degeneration. ROS and reactive nitrosative species (RNS) generated by UV can damage critical cellular components (DNA, proteins, lipids) and also affect the regulation of signaling molecules, such as mitogen-activated protein kinases (MAPKs), inflammatory cytokines, as well as nuclear factor-kβ (NF-κβ) and activator protein-1 (AP-1). Furthermore, photo-exposure induces the activation of the enzymatic systems, e.g., lipoxygenase (LOX) and cyclooxygenase (COX), which are responsible for the production of additional inflammatory mediators. It is also well established that cutaneous exposure to UV radiation contributes to the development of skin cancers. Epidemiological, clinical and pre-clinical studies have shown that solar UV radiation is the major etiological factor in the development of cutaneous malignancy [46], including the non-melanoma skin cancers, which represent the most common malignant neoplasms in humans [47]. Various animal models have been employed to examine the anti-photocarcinogenic effects of plant polyphenols. The plant polyphenols possess anti-inflammatory, immunomodulatory, antioxidant properties and DNA repair activities, and they can be exploited for the prevention of a variety of skin disorders caused by excessive exposure to solar UV light. *In vitro* and *in vivo* systems have both shown the protective effects of polyphenols on the biochemical processes that are induced or mediated by UV radiation, suggesting that routine use of natural polyphenols both topically and orally may provide effective protection against UV radiation, and subsequent photoaging [48]. Cellular studies and results from oral treatment and topical application studies in both animals and humans provide evidence that cocoa polyphenols, especially those belonging to the flavanol family, can offer effective photoprotection. Furthermore, the antioxidant and anti-inflammatory properties of cocoa polyphenols may constitute the basis of the possible antitumor promoting effects of these phytochemicals. A study conducted in rodents has demonstrated that a high phenolic extraction from cocoa powder (containing 468 mg/g of gallic acid-equivalent phenolics and 413 mg/g epicatechin-equivalent flavonoids) strongly inhibits the induction of COX-2 expression, the activation of MAPKs and NF-κβ signaling in 12-O-tetradecanoylphorbol-13-acetate (TPA)-treated mouse skin [49]. In particular, oral administering of cocoa polyphenols (4, 20, 40 and 200 mg/kg body weight) to mice 1 h prior to TPA exposure (10 nmol) inhibited ear edema at 5 h in a dose-dependent manner. The levels of COX-2 expression induced in mouse skin after 4-h treatment with topical TPA (10 nmol) was also diminished significantly by pretreating with cocoa polyphenols (40 or 200 mg/kg) for 30 min [49]. A more recent study has demonstrated that cocoa polyphenol extract effectively inhibited TNF-α-induced vascular endothelial growth factor (VEGF) expression in mouse epidermal cells [50]. This effect has been related to the ability of cocoa polyphenols to block TNF-α-induced activation of the nuclear transcription factors, AP-1 and NF-κβ, which are key regulators of VEGF expression. Other studies have explored the protective action of cocoa directly applied on the skin. The topical application of cocoa polyphenols has been shown to positively affect several parameters of skin elasticity and skin tonus, namely, glycosaminoglycans and collagen I, III and IV [51]. Moreover, topical application of both cocoa extract titled in theobromine or pure theobromine have been shown to prevent UV-induced wrinkle formation in hairless mice. Mice were exposed to solar simulated ultraviolet irradiation at a dose of 13.0 J/cm^2^ (UVA) for 15 weeks, five times a week on weekdays. After the final irradiation, histological and analytical studies showed that the topical application of cacao extracts or theobromine markedly prevented photodamage, including wrinkle formation, dermal connective alteration and collagen accumulation. The research also suggested that xanthine derivatives prevented neutrophil infiltration caused by UV-irradiation, supporting a critical role for theobromine in the dermal protective action of cocoa [52]. *In vivo* human skin studies have also shown the strong anti-inflammatory, antioxidant, photoprotective and chemopreventative effects of cocoa, after oral consumption. In a double-blind clinical trial, two groups of women, with healthy and normal skin of type II, consumed either a high flavanol (326 mg/day) or low flavanol (27 mg/day) cocoa powder dissolved in 100 mL water for 12 weeks. Epicatechin (61 mg/day) and catechin (20 mg/day) were the major flavanol monomers in the high flavanol drink, whereas the low flavanol drink contained 6.6 mg epicatechin and 1.6 mg catechin as the daily dose. Dietary intervention with a cocoa beverage rich in flavanols decreased the sensitivity of human skin toward UV light, which was determined by the degree of erythema (reddening) following irradiation with a solar light simulator. Compared to baseline, skin response was decreased by 15% after six weeks of intervention, and the decrease was more pronounced, 25%, after 12 weeks. UV sensitivity did not change in the women that consumed the cocoa beverage low in flavanols. Compared to the low flavanol product, the high flavanol cocoa powder contained ~10× the amount of epicatechin and catechin, as well as total flavanols. The same study has found an increase in cutaneous and subcutaneous blood flow in women supplemented for 12 weeks with a cocoa beverage rich in flavanols. At Week 12, blood flow increased ~100% at a 1-mm depth and ~40% at a 7–8-mm depth compared with baseline [19]. Microcirculation is an important factor for thermoregulation, nutrient and oxygen supply, and it affects skin condition and appearance [53]. To better assess the activity of flavanol-rich cocoa acute consumption on dermal blood flow, in a crossover study, 10 healthy women ingested a cocoa drink (100 mL) with a high (329 mg) or a low (27 mg) content of flavanols. Dermal blood flow and oxygen saturation of hemoglobin were examined by laser Doppler flowmetry and spectroscopically at a 1-mm skin depth at *t* = 0, 1, 2, 4 and 6 h. At the same time points, plasma levels of total epicatechin (free compound plus conjugates) were measured by means of HPLC. Subsequent to the intake of high flavanol cocoa, dermal blood flow was significantly increased by 1.7-fold at *t* = 2 h, and oxygen saturation was elevated 1.8-fold. No statistically significant changes were found upon the intake of low flavanol cocoa. Maximum plasma levels of total epicatechin were observed 1 h after ingestion of the high flavanol cocoa drink, 11.6 ± 7.4 nmol/L at baseline and 62.9 ± 35.8 nmol/L at 1 h [20]. The relationship between dark chocolate consumption rich in flavanols and skin photoprotection has been investigated in a double-blind *in vivo* study in 30 healthy subjects. Fifteen subjects each were randomly assigned to either a high flavanol or low flavanol chocolate group and consumed a 20-g portion of their allocated chocolate daily. The minimal erythema dose (MED) was assessed at baseline and after 12 weeks under standardized conditions. In the high flavanol chocolate group, the mean MED more than doubled after 12 weeks of chocolate consumption, while in the low flavanol chocolate group, the MED remained without significant change. These results demonstrated that regular consumption of a chocolate rich in flavanols confers significant photoprotection and can thus be effective at protecting human skin from harmful UV effects [54]. Although more clinical evidence is probably needed to better evaluate the clinical relevance of polyphenols in dermatology, the data presented above clearly showed that cocoa components have important antioxidant, anti-inflammatory and photoprotective functions on the skin. The ability of cocoa phytochemicals to modulate critical biochemical functions through oral and topical formulations makes cocoa a promising candidate for further dermatological applications, ranging from cosmetic wellness to the prevention of carcinogenesis.

## 5. Conclusions

Cocoa and cocoa products are important sources of phytocompounds with nutritional and therapeutic value. A growing body of scientific evidence is becoming available to support that cocoa components with antioxidants and anti-inflammatory activities contribute to endogenous photoprotection and are crucial for the maintenance of skin health. However, several studies have shown that the beneficial effects of cocoa vary among the wide range of cocoa and chocolate products. Various mechanisms have been proposed to explain the possible benefits of cocoa consumption on human health, but a clear molecular mechanism is still lacking. Although clinical data provide critical evidence of the health value of cocoa, the main cellular processes and signaling pathways involved in the detoxification of reactive species and the removal of pro-inflammatory mediators are significantly underestimated. Moreover, since cocoa contains a mixture of bioactive components, further studies are needed to determine the possible synergistic interaction between them. For several reasons, cocoa-derived phytochemicals are receiving increasing interest from consumers and food manufacturers, but the bioavailability, the kinetics of absorption and the bioactivity are not well-established. Pertaining to skin health, cocoa components have been utilized in diseases, such as skin cancer, psoriasis, acne and wound healing. It is noteworthy that is has been shown that cocoa has great potential not only for the treatments of skin diseases, but also for their prevention. In particular, antioxidants found in cocoa protect the skin from the inside by neutralizing oxidative stress, a major factor of dermal structure deterioration and premature skin aging. In conclusion, multiple lines of evidence support the role of cocoa in the promotion of human health, but a full understanding of the mechanisms of action of cocoa-derived phytochemicals as modulators of cell signaling is the key to evaluate the efficiency of these potent biomolecules as anti-aging agents.

## References

[1] Dillinger T.L., Barriga P., Escárcega S., Jimenez M., Salazar Lowe D., Grivetti L.E. (2000). Food of the gods: Cure for humanity? A cultural history of the medicinal and ritual use of chocolate. J. Nutr..

[2] Tomaru M., Takano H., Osakabe N., Yasuda A., Inoue K., Yanagisawa R., Ohwatari T., Uematsu H. (2007). Dietary supplementation with cacao liquor proanthocyanidins prevents elevation of blood glucose levels in diabetic obese mice. Nutrition.

[3] Hurst W.J., Krake S.H., Bergmeier S.C., Payne M.J., Miller K.B., Stuart D.A. (2011). Impact of fermentation, drying, roasting and Dutch processing on flavan-3-ol stereochemistry in cacao beans and cocoa ingredients. Chem. Cent. J..

[4] Miller K.B., Hurst W.J., Payne M.J., Stuart D.A., Apgar J., Sweigart D.S., Ou B. (2008). Impact of alkalization on the antioxidant and flavanol content of commercial cocoa powders. J. Agric. Food Chem..

[5] Ellam S., Williamson G. (2013). Cocoa and human health. Annu. Rev. Nutr..

[6] Solà R., Valls R.M., Godàs G., Perez-Busquets G., Ribalta J., Girona J., Heras M., Cabré A., Castro A., Domenech G. (2012). Cocoa, hazelnuts, sterols and soluble fiber cream reduces lipids and inflammation biomarkers in hypertensive patients: A randomized controlled trial. PLoS One.

[7] Corti R., Flammer A.J., Hollenberg N.K., Lüscher T.F. (2009). Cocoa and cardiovascular health. Circulation.

[8] Lee K.W., Kim Y.J., Lee H.J., Lee C.Y. (2003). Cocoa has more phenolic phytochemicals and a higher antioxidant capacity than teas and red wine. J. Agric. Food Chem..

[9] Katz D.L., Doughty K., Ali A. (2011). Cocoa and chocolate in human health and disease. Antioxid. Redox Signal..

[10] Kim J., Kim J., Shim J., Lee C.Y., Lee K.W., Lee H.J. (2014). Cocoa phytochemicals: Recent advances in molecular mechanisms on health. Crit. Rev. Food Sci. Nutr..

[11] Manach C., Scalbert A., Morand C., Rémésy C., Jiménez L. (2004). Polyphenols: Food sources and bioavailability. Am. J. Clin. Nutr..

[12] Harborne J.B., Williams C.A. (2000). Advances in flavonoid research since 1992. Phytochemistry.

[13] Bravo L. (1998). Polyphenols: Chemistry, Dietary Sources, Metabolism, and Nutritional Significance. Nutr. Rev..

[14] Cheynier V. (2005). Polyphenols in foods are more complex than often thought. Am. J. Clin. Nutr..

[15] Tsao R. (2010). Chemistry and biochemistry of dietary polyphenols. Nutrients.

[16] Ortega N., Romero M.P., Macià A., Reguant J., Anglès N., Morelló J.R., Motilva M.J. (2008). Obtention and characterization of phenolic extracts from different cocoa sources. J. Agric. Food Chem..

[17] Ramiro-Puig E., Castell M. (2009). Cocoa: Antioxidant and immunomodulator. Br. J. Nutr..

[18] Jalil A.M., Ismail A. (2008). Polyphenols in cocoa and cocoa products: Is there a link between antioxidant properties and health?. Molecules.

[19] Heinrich U., Neukam K., Tronnier H., Sies H., Stahl W. (2006). Long-term ingestion of high flavanol cocoa provides photoprotection against UV-induced erythema and improves skin condition in women. J. Nutr..

[20] Neukam K., Stahl W., Tronnier H., Sies H., Heinrich U. (2007). Consumption of flavanol-rich cocoa acutely increases microcirculation in human skin. Eur. J. Nutr..

[21] Ashihara H., Kato M., Crozier A. (2011). Distribution, biosynthesis and catabolism of methylxanthines in plants. Handb. Exp. Pharmacol..

[22] Smit H.J., Blackburn R.J. (2005). Reinforcing effects of caffeine and theobromine as found in chocolate. Psychopharmacology (Berl).

[23] Smit H.J., Gaffan E.A., Rogers P.J. (2004). Methylxanthines are the psycho-pharmacologically active constituents of chocolate. Psychopharmacology (Berl).

[24] Neufingerl N., Zebregs Y.E., Schuring E.A., Trautwein E.A. (2013). Effect of cocoa and theobromine consumption on serum HDL-cholesterol concentrations: A randomized controlled trial. Am. J. Clin. Nutr..

[25] Blinks J.R., Olson C.B., Jewell B.R., Bravený P. (1972). Influence of caffeine and other methylxanthines on mechanical properties of isolated mammalian heart muscle. Evidence for a dual mechanism of action. Circ. Res..

[26] Shively C.A., Tarka S.M. (1984). Methylxanthine composition and consumption patterns of cocoa and chocolate products. Prog. Clin. Biol. Res..

[27] Sprügel W., Mitznegg P., Heim F. (1977). The influence of caffeine and theobromine on locomotive activity and the brain cGMP/cAMP ratio in white mice. Biochem. Pharmacol..

[28] Scapagnini G., Davinelli S., Drago F., de Lorenzo A., Oriani G. (2012). Antioxidants as antidepressants: Fact or fiction?. CNS Drugs.

[29] Judelson D.A., Preston A.G., Miller D.L., Muñoz C.X., Kellogg M.D., Lieberman H.R. (2013). Effects of theobromine and caffeine on mood and vigilance. J. Clin. Psychopharmacol..

[30] Scholey A., Owen L. (2013). Effects of chocolate on cognitive function and mood: A systematic review. Nutr. Rev..

[31] F’guyer S., Afaq F., Mukhtar H. (2003). Photochemoprevention of skin cancer by botanical agents. Photodermatol. Photoimmunol. Photomed..

[32] Steinberg F.M., Bearden M.M., Keen C.L. (2003). Cocoa and chocolate flavonoids: implications for cardiovascular health. J. Am. Diet. Assoc..

[33] Gums J.G. (2004). Magnesium in cardiovascular and other disorders. Am. J. Health Syst. Pharm..

[34] Ueshima K. (2005). Magnesium and ischemic heart disease: A review of epidemiological, experimental, and clinical evidences. Magnes. Res..

[35] Olivares M., Uauy R. (1996). Copper as an essential nutrient. Am. J. Clin. Nutr..

[36] Kris-Etherton P.M., Keen C.L. (2002). Evidence that the antioxidant flavonoids in tea and cocoa are beneficial for cardiovascular health. Curr. Opin. Lipidol..

[37] Serafini M., Bugianesi R., Maiani G., Valtuena S., de Santis S., Crozier A. (2003). Plasma antioxidants from chocolate. Nature.

[38] Engler M.B., Engler M.M. (2006). The emerging role of flavonoid-rich cocoa and chocolate in cardiovascular health and disease. Nutr. Rev..

[39] Scapagnini G., Vasto S., Abraham N.G., Caruso C., Zella D., Fabio G. (2011). Modulation of Nrf2/ARE pathway by food polyphenols: A nutritional neuroprotective strategy for cognitive and neurodegenerative disorders. Mol. Neurobiol..

[40] Shah Z.A., Li R.C., Ahmad A.S., Kensler T.W., Yamamoto M., Biswal S., Doré S. (2010). The flavanol (−)-epicatechin prevents stroke damage through the Nrf2/HO1 pathway. J. Cereb. Blood Flow Metab..

[41] Schroeter H., Heiss C., Balzer J., Kleinbongard P., Keen C.L., Hollenberg N.K., Sies H., Kwik-Uribe C., Schmitz H.H., Kelm M. (2006). (−)-Epicatechin mediates beneficial effects of flavanol-rich cocoa on vascular function in humans. Proc. Natl. Acad. Sci. USA.

[42] Sies H., Schewe T., Heiss C., Kelm M. (2005). Cocoa polyphenols and inflammatory mediators. Am. J. Clin. Nutr..

[43] Lee K.W., Kang N.J., Oak M.H., Hwang M.K., Kim J.H., Schini-Kerth V.B., Lee H.J. (2008). Cocoa procyanidins inhibit expression and activation of MMP-2 in vascular smooth muscle cells by direct inhibition of MEK and MT1-MMP activities. Cardiovasc. Res..

[44] Ramiro E., Franch A., Castellote C., Pérez-Cano F., Permanyer J., Izquierdo-Pulido M., Castell M. (2005). Flavonoids from Theobroma cacao down-regulate inflammatory mediators. J. Agric. Food Chem..

[45] Selmi C., Mao T.K., Keen C.L., Schmitz H.H., Eric Gershwin M. (2006). The anti-inflammatory properties of cocoa flavanols. J. Cardiovasc. Pharmacol..

[46] Afaq F., Adhami V.M., Mukhtar H. (2005). Photochemoprevention of ultraviolet B signaling and photocarcinogenesis. Mutat. Res..

[47] Urbach F. (1991). Incidence of nonmelanoma skin cancer. Dermatol. Clin..

[48] Nichols J.A., Katiyar S.K. (2010). Skin photoprotection by natural polyphenols: Anti-inflammatory, antioxidant and DNA repair mechanisms. Arch. Dermatol. Res..

[49] Lee K.W., Kundu J.K., Kim S.O., Chun K.S., Lee H.J., Surh Y.J. (2006). Cocoa polyphenols inhibit phorbol ester-induced superoxide anion formation in cultured HL-60 cells and expression of cyclooxygenase-2 and activation of NF-kappaB and MAPKs in mouse skin *in vivo*. J. Nutr..

[50] Kim J.E., Son J.E., Jung S.K., Kang N.J., Lee C.Y., Lee K.W., Lee H.J. (2010). Cocoa polyphenols suppress TNF-α-induced vascular endothelial growth factor expression by inhibiting phosphoinositide 3-kinase (PI3K) and mitogen-activated protein kinase kinase-1 (MEK1) activities in mouse epidermal cells. Br. J. Nutr..

[51] Gasser P., Lati E., Peno-Mazzarino L., Bouzoud D., Allegaert L., Bernaert H. (2008). Cocoa polyphenols and their influence on parameters involved in *ex vivo* skin restructuring. Int. J. Cosmet. Sci..

[52] Mitani H., Ryu A., Suzuki T., Yamashita M., Arakane K., Koide C. (2007). Topical application of plant extracts containing xanthine derivatives can prevent UV-induced wrinkle formation in hairless mice. Photodermatol. Photoimmunol. Photomed..

[53] Braverman I.M. (2000). The cutaneous microcirculation. J. Investig. Dermatol. Symp. Proc..

[54] Williams S., Tamburic S., Lally C. (2009). Eating chocolate can significantly protect the skin from UV light. J. Cosmet. Dermatol..

